# Serum levels of interleukin-6 are not dependent on the kidney function

**DOI:** 10.1155/S0962935192000309

**Published:** 1992

**Authors:** L. Nässberger

**Affiliations:** Department of Medical Microbiology Clinical Immunology Lund University Sölvegatan 23 Lund 223 62 Sweden

## Abstract

Interleukin-6, also named B-cell stimulatory factor, is a glycoprotein with a molecular weight of 26 kDa. Increased serum levels of interleukin-6 (IL-6) are found in several disease conditions. We investigated the importance of a deteriorated kidney function upon IL-6 serum concentrations. No relation was found between serum levels of IL-6 and s-creatinine, r = 0.004. On the other hand, the serum concentration of complement protein factor D and soluble IL-2 receptor showed a good correlation to s-creatinine, r = 0.92 and 0.79, respectively. In conclusion, serum levels of IL-6 are not dependent upon a reduced kidney function.


Mediators of Inflammation, 1, 197–199 (1992)

### Serum levels of interleukin-6 are not dependent on the kidney function

L. Nässberger

Department of Medical Microbiology, Clinical Immunology, Lund University, Sölvegatan 23, 223 62 Lund, Sweden

**INTERLEUKIN-6, also named B-cell stimulatory factor, is a glycoprotein with a molecular weight of 26 kDa. Increased serum levels of interleukin-6 (IL-6) are found in several disease conditions. We investigated the importance of a deteriorated kidney function upon IL-6 serum concentrations. No relation was found between serum levels of IL-6 and s-creatinine, $r = 0.004$. On the other hand, the serum concentration of complement protein factor D and soluble IL-2 receptor showed a good correlation to s-creatinine, $r = 0.92$ and 0.79, respectively. In conclusion, serum levels of IL-6 are not dependent upon a reduced kidney function.**

**Key words:** Interleukin-6, Kidney, Low molecular weight protein

### Introduction

Interleukins or cytokines are message molecules which regulate important immunological functions. All interleukins have low molecular weights. Increased levels of interleukin-6 (IL-6), also known as the B-cell stimulatory factor, or originally named interferon $\beta_2$ (IFN$\beta_2$) are found after a variety of tissue responses ranging from minor stress,[1] elective surgery,[2] severe sepsis,[3] and in bacterial peritonitis in patients undergoing continuous ambulatory peritoneal dialysis, CAPD.[4] IL-6 seems to be produced in most nuclear containing cells. It appears to be one of the major mediators of the reaction to viral and bacterial infections, inflammation and shock.[5] It has also been experienced that the level of IL-6 increases earlier than acute proteins such as C-reactive protein and $\alpha$1-antitrypsin.

IL-6 is a protein with 184 amino acids and a molecular weight of about 26 kDa, so it can be classified as a low molecular weight protein.[6] Therefore, it can be filtered through the glomeruli. A reduced kidney function could be assumed to increase serum IL-6 levels. Increased serum levels of small molecular weight proteins i.e. $\beta$-microglobulin and factor D are seen in patients with reduced glomerular filtration rate.[7,8] The aim of this study was to investigate the influence of kidney function upon serum levels of IL-6.

### Materials and Methods

*Patients:* Twenty-one patients with adult polycystic kidney disease were included in the study. Except for their kidney disease they were all well and there were no signs of an ongoing infection, or other diseases. Serum creatinine values ranged from 80 to 1390 $\mu$mol/l.

*Normal sera:* Sera from 25 normal blood donors served as control for establishing the cut-off level in the IL-6 assay.

*Methods:* Serum creatinine were measured by a conventional technique at our department of clinical chemistry. Factor D was measured by a haemolysis technique.[9] The concentration is given as a percentage of normal sera. Soluble interleukin-2 receptor (S-IL-2R) was measured using an ELISA method (T-cell Science, Boston). Briefly, wells were coated with a murine monoclonal antibody directed against an epitope on the S-IL-2R molecule. A horseradish peroxidase conjugated monoclonal antibody directed against another epitope on S-IL-2 is used as the detecting (secondary) antibody.

*IL-6 assay:* Serum interleukin-6 (IL-6) was assayed using an ELISA method (Innogenetics A.S., Antwerp, Belgium). Briefly, polystyrene microplates were coated with sheep polyclonal anti-IL-6 antibodies. One hundred microlitres of the respective samples were incubated for 2 h at 37°C. A murine monoclonal anti-IL-6 biotin-labelled antibody was then added, followed by peroxidase conjugated streptavidin. The substrate used was tetramethylbenzidine and the developed colour was read at 450 nm.

### Results

*Normal sera:* The mean absorbance, when subtracting the background values, was 0.058. This value corresponds to a concentration of 10 pg/ml, when reading from the standard curve. The cut-off level was set at 10 pg/ml, which corresponds to the recommendation given by the manufacturer.

© 1992 Rapid Communications of Oxford Ltd

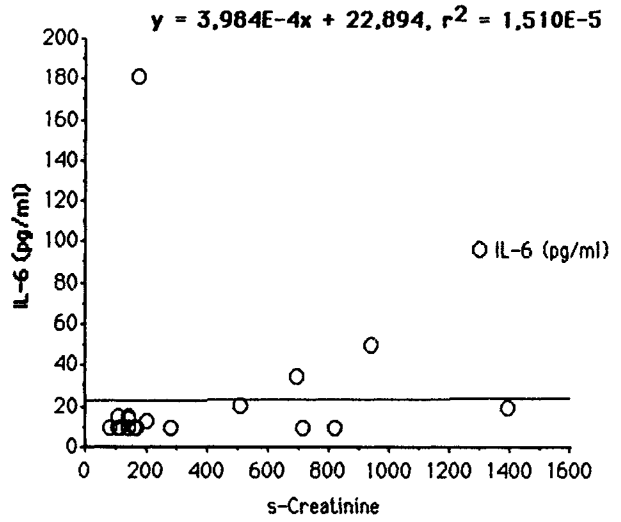

FIG. 1. Shows the relation between serum concentration of IL-6 and s-creatinine. The correlation coefficient was $r = 0.0004$.

*Patients:* Twelve of the patients had serum concentrations of IL-6 below 10 pg/ml. One patient had a very high serum concentration, 180 pg/ml. As seen in Figure 1 no correlation was seen between S-IL-6 and s-creatinine ($r = 0.004$). There was no correlation between serum levels of IL-6 and levels of factor D and S-IL-2R. The correlation coefficients were $r = 0.028$ and $r = 0.018$, respectively. On the other hand, a very high correlation was found between Factor D and s-creatinine as seen in Figure 2a ($r = 0.92$; $p = 0.0001$). A somewhat lower relation was observed between S-IL-2R and s-creatinine ($r = 0.79$; $p = 0.001$) (Figure 2b).

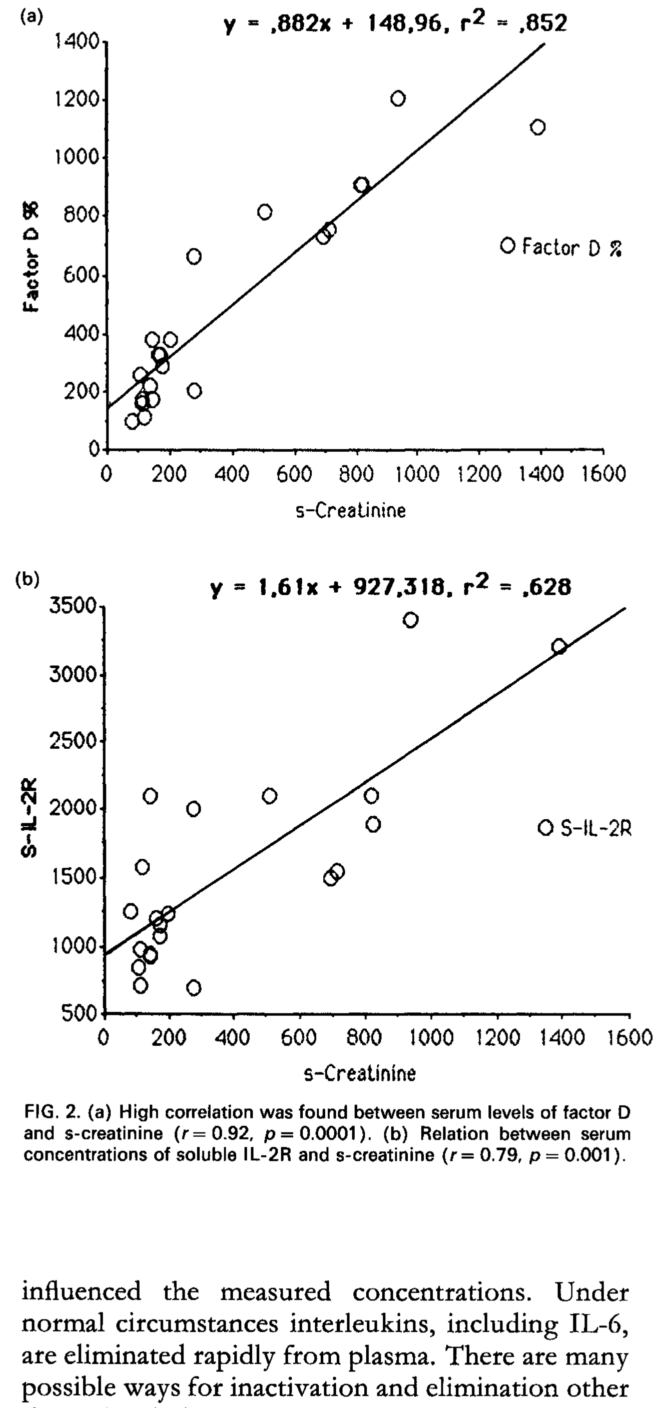

FIG. 2. (a) High correlation was found between serum levels of factor D and s-creatinine ($r = 0.92$, $p = 0.0001$). (b) Relation between serum concentrations of soluble IL-2R and s-creatinine ($r = 0.79$, $p = 0.001$).

### Discussion

IL-6 is a low molecular weight protein synthesized in monocytes, though it can be produced and secreted from all nuclear cells. It was first identified by its ability to induce antibody secretion by preactivated normal and Epstein—Barr virus transformed human B-cells[10] without first inducing cellular proliferation. Apart from this effect, it has been found that IL-6 induces differentiation of cytotoxic T-cells from both mature and immature T-cells.[11]

Several lines of evidence suggest that IL-6 is involved in the pathogenesis of certain autoimmune diseases.[12] High levels have been found in synovial fluid in rheumatoid patients, but not in those with active osteoarthritis. Furthermore, increased serum levels of IL-6 have been found during allograft rejection.[13] A close relation is also seen between body temperature and IL-6 levels.[14] Since serum levels of IL-6 are increased in several different diseases, it could be of great importance to investigate how a reduced kidney function influenced the measured concentrations. Under normal circumstances interleukins, including IL-6, are eliminated rapidly from plasma. There are many possible ways for inactivation and elimination other than the kidneys, for instance inactivation by proteases,[15] binding to circulating soluble receptors such as soluble TNF receptor,[16] to carriers such as $\alpha_2$-macroglobulin,[17] or to autoantibodies.[18] This study shows clearly that a reduced kidney function does not have a major effect on the serum levels of IL-6. Therefore, measured levels are not falsely high values recorded due to a deteriorated kidney function, but reflect an inflammatory response *per se*. These findings indicate that kidneys do not contribute to any great extent to the clearance of IL-6. The same condition seems to be valid for interleukin-1. Radiolabelled IL-1 was injected to nephrectomized rats, and there was only a small increase of serum IL-1 levels in these rats compared to non-nephrectomized rats. The authors concluded that less than 10% of IL-1 was cleared by the kidney.[19] Though renal catabolism may play a role, and tubular cells have been found to reabsorb interleukins, (cf. Ref. 20) other elimination pathways may account for an optimal clearance. In patients with renal dysfunction it could be assumed that some or several of the above mentioned pathways are stimulated, leading to an enhancement of inactivation of cytokines.

Factor D is a complement protein and the S-IL-2R is a T-lymphocyte derived product with a molecular weight of 45 kDa compared to 23.5 kDa for Factor D. Serum levels for both these proteins depend on kidney function and high correlation coefficients to s-creatinine were found. Factor D was seen to correlate even better for s-creatinine than S-IL-2R. Recently it has also been shown that circulating soluble tumour necrosis factor binding protein is highly correlated to s-creatinine[21] in a manner similar to S-IL-2R.

The high level of IL-6 in one of the patients was puzzling. At the time of sampling there was no sign of infection or inflammatory state. It is known that IL-6 can be produced locally in the kidney due to hypercellularity, for instance in renal cell carcinoma[22] or in mesangial proliferative glomerulonephritis.[23] It has also recently been shown that other tumours may cause IL-6 production, for instance myeloma and cardiac myxoma cells. It was not completely ruled out that our patient might have had a tumour, but at the time of sampling no indication of neoplasm or other disorder was observed. Follow-up has, however, not been possible. It has been demonstrated that *in vivo* administration of recombinant human tumour necrosis factor to patients with metastatic cancer leads to the induction of circulating levels of IL-6.[24] From an experimental point of view it has also been shown that tumour necrosis factor influences IL-6 kinetics.[25]

In conclusion, when evaluating serum levels of IL-6 in different disease states there is no need for correction due to a reduced glomerular filtration rate.

### References

1. LeMay LG, Vander AJ, Kluger MJ. The effects of physiological stress on plasma interleukin-6 activity in rats. *Physiol Behav* 1990; **47**: 957–961.
2. Nishimoto N, Yoshizaki K, Tagoh H, *et al.* Elevation of serum interleukin-6 prior to acute phase proteins on the inflammation by surgical operation. *Clin Immunol Immunopathol* 1989; **50**: 399–401.
3. Hach CE, Degroot ER, Feltbersma RJF, *et al.* Increased plasma levels of Interleukin-6 in sepsis. *Blood* 1989; **74**: 1704–1710.
4. Metsärinne K, Teppo AM, Grönhagen-Riska C, Fyhrquist F. Increased plasma Interleukin-6 and renin substrate levels during bacterial peritonitis in CAPD patients. *Clin Nephrol* 1991; **36**: 104.
5. Wong GG, Clark SC. Multiple actions of interleukin-6 within a cytokine network. *Immunol Today* 1988; **9**: 137–139.
6. van Damme J, Opdenakker G, Simpson RJ, *et al.* Identification of the human 26 kDa protein, interferon$\beta$2 (IFN-$\beta$2), as a B cell hybridoma/plasmacytoma growth factor induced by interleukin-1 and tumour necrosis factor. *J Exp Med* 1987; **165**: 914–919.
7. Wibell L, Evrin PE, Berggård I. Serum $\beta$2-microglobulin in renal disease. *Nephron* 1973; **10**: 320–331.
8. Sturfelt G, Truedsson L, Thysell H, Björck S. Serum level of complement factor D in systemic lupus erythematosus—an indicator of glomerular filtration rate. *Acta Med Scand* 1984; **216**: 171–177.
9. Martin H, Lackman PJ, Halbwachs L, Hobart MJ. Haemolytic diffusion plate assays for factors B and D of the alternative pathway of complement activation. *Immunochemistry* 1976; **13**: 317–324.
10. Hiravio T, Yasukawa K, Harada H, *et al.* Complementary DNA for a novel human interleukin (BSF-2) that induces B lymphocytes to produce immunoglobulin. *Nature* 1986; **324**: 73–76.
11. Uyttenhove C, Coulie PG. van Snick J. T cell growth and differentiation induced by interleukin HPI/IL-6, the murine hybridoma plasmacytoma growth factor. *J Exp Med* 1988; **167**: 1417–1427.
12. Tovey MG, Gresser I, Blanchard B, Guymark OJ. Expression of IL-6 in normal individuals and in patients with autoimmune disease. In: Sehgal B, Grieninger G, Tosato G, eds. *Regulation of the acute and immune responses, Interleukin-6. Annals NY Acad Sci* 1989; **557**: 363–373.
13. van Oers MHK, van Der Heyden PAM, Aarden LA. Interleukin-6 (IL-6) in serum and urine of renal transplant recipients. *Clin Exp Immunol* 1988; **71**: 314–319.
14. Nijsten MWN, De Groot ER, Tenduis HJ, Klasen HJ, Hack LE, Aarden LA. Serum levels of Interleukin-6 and acute phase responses. *Lancet* 1987; **ii**: 921.
15. Konrad MW, Hemstreet G, Hersh EM, *et al.* Pharmacokinetics of recombinant Interleukin-2 in humans. *Cancer Research* 1990; **50**: 2009–2017.
16. Seckinger P, Zhang JH, Hauptman B, Dayer JM. Characterization of a tumour necrosis factor $\alpha$ (TNF-$\alpha$) inhibitor, evidence of immunological cross-reactivity with the TNF-receptor. *Proc Natl Acad Sci USA* 1990; **87**: 5188–5192.
17. James K. Interactions between cytokines and $\alpha_2$ macroglobulin. *Immunology Today* 1990; **11**: 163–166.
18. Bendtzen K, Svenson M, Jonsson V, Hippe E. Autoantibodies to cytokines: friends or foes. *Immunology Today* 1990; **11**: 167–169.
19. Kampschmidt RF, Jones T. Rate of clearance of Interleukin-1 from the blood of normal and nephrectomized rats. *Proc Soc Exp Biol Med* 1985; **180**: 170–173.
20. Bocci V. Interleukins, clinical pharmacokinetics and practical implications. *Clin Pharmacokinet* 1991; **21**: 274–284.
21. Lantz M, Thysell H, Nilsson E, Olsson I. On the binding of tumour necrosis factor (TNF) to heparin and the release *in vivo* of the TNF-binding protein-1 by heparin. *J Clin Invest* 1991; **88**: 2026–2031.
22. Miki S, Iwano M, Miki Y, *et al.* Interleukin-6 (IL-6) functions as an *in vitro* autocrine growth factor in renal cell carcinomas. *FEBS Lett* 1989; **250**: 607–610.
23. Gordon C, Richards N, Howie AJ, *et al.* Urinary IL-6: a marker for mesangial proliferative glomerulonephritis. *Clin Exp Immunol* 1991; **86**: 145–149.
24. Jablons DM, Mulé JJ, McIntosh JK, *et al.* Interleukin-6/interferon-$\beta$2 as a circulating hormone: induction by cytokine administration in humans. *J Immunol* 1989; **142**: 1542–1545.
25. McIntosh IK, Mulé JJ, Jablons MJ, *et al.* The kinetics of Interleukin-6 induction by the systemic administration of rhTNF-$\alpha$ in mice. In Sehgal B, Grieninger G, Tosato G. Regulation of the acute phase and immune responses Interleukin-6. *Annals NY Acad Sci* 1989; **557**: 572–575.

ACKNOWLEDGEMENTS. This study was supported by grants from the Association for Kidney Diseases, King Gustav V's 80th Birthday Fund, the Medical Faculty, Lund University, Kungl, Fysiografiska Sällskapet, Lund. The author is grateful to Mrs Britt-Marie Karlsson for skilful preparation of the manuscript.

**Received 3 March 1992; accepted in revised form 3 April 1992**
